# Managing demand generation with evidence

**Published:** 2019-12-17

**Authors:** Ramasamy Meenakshi Sundaram, Thulasiraj Ravilla, B.S. Ganesh Babu

**Affiliations:** 1 Senior Manager: Clinical Services, Aravind Eye Hospital, Madurai, India.; 2 Director of Operations: Aravind Eye Care System.; 3 Senior Manager: IT, Aravind Eye Care System.

**Generating and managing demand for eye care services must be based on evidence. We discuss what this evidence includes and how it can be effectively used to start eye care programmes to reach underserved populations.**

**Figure:**
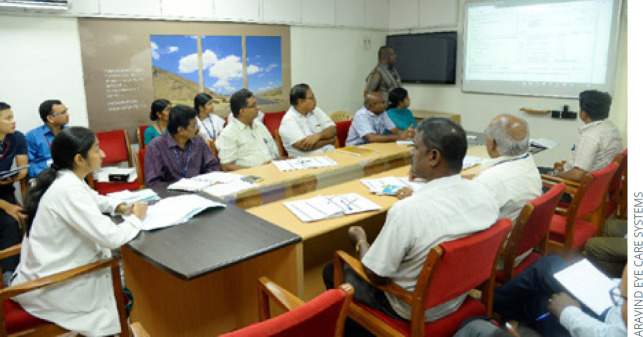
Evidence is needed to generate and manage demand for eye care services. INDIA

An estimated 1.3 billion people worldwide live with some form of distance or near vision impairment. Uncorrected refractive errors and cataracts are the leading causes of vision impairment. They are also avoidable.^1^ The WHO estimates that globally only about a quarter of people with eye problems use eye care services.^2^

Studies conducted in rural India and Nepal show the levels of uptake of eye services and cataract surgery range from seven per cent to 35 per cent. In another study in 52 countries only 18 per cent of people over 60 years got their eyes examined within the last one year; 38 per cent reported never having an eye exam.^3^

Appropriate demand generation strategies can address this unmet need in the community. With large eye care providers based in urban areas, there is a need to reach the rural population. Community outreach programmes are a viable strategy to generate demand in rural populations. These programmes have an indirect or snowball effect on the patients who come to a base hospital.^4^ Another strategy is setting up primary eye care centres in rural areas. Primary eye care centres can enable access and refer those who need further interventions to a base hospital.

## Outreach

Outreach can be either at a community screening or at vision centre or at primary eye care centre. An eye care provider can generate demand for outreach activities by gaining a good understanding of the region, and ensuring good frequency of visits. While walk-in patients can visit a hospital anytime, their visits depend on:

- their level of awareness
- the priority they accord to eye care and
- the level of trust in the service provider

We need to plan activities and track them with appropriate evidence to ensure that our efforts in delivering eye care are effective.

## Estimating the demand potential

While evidence exists for prevalence or backlog, it is a challenge to estimate the demand for, let's say, cataract surgeries or spectacles, from a particular area in a certain period. Yet, one can make reasonable estimates based on existing trends, comparing with other similar regions, and the need in the community. Such estimates can also be made for a country or state.

### Setting targets for an eye hospital

Besides, estimating the demand potential, eye hospitals needs to set targets which requires us to understand the current level of eye care in the service area to arrive at the unmet demand potential. Targets combined with the hospital's own capacity can help derive the annual goals for the hospital.

### Setting targets for an outreach event

An outreach event covers a circle of eight to ten kilometres radius around the camp and is influenced by the access time to reach the eye camp site. The population covered and the intensity of promotion are the main drivers of the event. Past experiences help to refine the estimates for expected outpatients, surgery or spectacles.

### Why are such targets important?

At the national level such targets are critical for advocacy and to build capacity of human resources and facilities. This applies to the hospital level as well. For outreach events such targets guide adequate staffing. Technology like geographic information system (GIS) can help to set realistic targets for served or underserved areas.

## Ensuring effectiveness of the programme

Several factors influence the effectiveness of our efforts or deployment of resources. Having evidence helps to develop the right interventions to enhance the effectiveness. Such factors are:

**Compliance** refers to whether or not patients follow their physicians instructions. It is important to understand how many patients comply with the prescribed surgery, spectacles or treatment. All the diagnostic or outreach efforts on those who don't comply, is essentially wasted. Plus without the treatment, there is no impact on the problem. So it is important to track the prescriptions and their compliance. Understanding the barriers to non-compliance helps to plan appropriate strategies to improve compliance.

**Figure:**
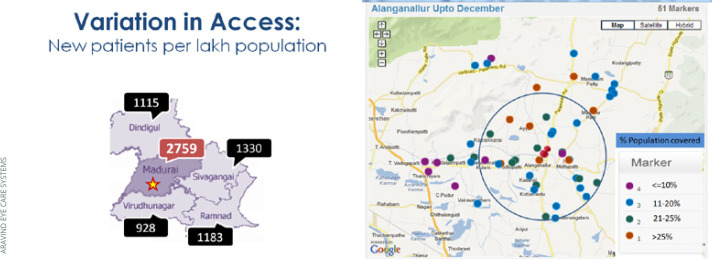
A geographical analysis can tell where patients come from. INDIA

**Cataract surgery acceptance rate** is the number of cataract patients accepting a surgery among the total number of patients prescribed. Setting a target for the surgery acceptance rate helps to measure the gap and plan for increased productivity and cost effectiveness. You may find that transport, counselling and costs are also facilitators for improving effectiveness.

**Cataract conversion rate** is the number of cataract surgeries done per hundred outpatients served. We use this when calculating cataract surgery acceptance rate is not possible due to lack of data. The rate varies across the regions as per the prevalence of cataract and the type of services available. Comparing the rate with similar organisations ensures that we do not miss the patients needing cataract surgery. The rate can suggest refinements to clinical protocol and counselling process.

**Diagnostic profile** of outpatients gives an insight into the patients' condition. We must take appropriate action if patients are not visiting the hospital for certain conditions. For instance, if very few patients with diabetes are getting their eyes examined, focused awareness campaigns at the community level can address this and reduce the risk of diabetic retinopathy.

A **geographic analysis** of where patients come from can help to identify areas of low coverage. This, in turn, helps to frame appropriate strategies to reach patients from all locations.

It is a common phenomenon in many regions to see seasonal variations in **patient load**. This can be due to several reasons, including changes in the weather. Such variations lead to underutilising resources during lean periods and stretching in peak periods. Both scenarios are undesirable. So, knowing the variations would help managers to smooth the seasonality.

## Sustaining the demand

Sustaining and growing the demand is largely driven by word of mouth and trust in the hospital. Therefore the evidence for quality of services, patient satisfaction and retention requires constant improvement.

### Post-operative visual outcome

Satisfied patients usually become the ambassadors of the hospital. It is important to minimise operative complications and ensure that the patient gets the best possible vision. The WHO recommends that over 90 per cent of the cataract surgical patients should gain a best corrected vision of 6/18 or better. It is heartening that very good outcome of better or equal to 6/12 is a step in the right direction for quality vision.

### Patient experience

This is another major influence on demand. It includes both clinical and non-clinical services. Apart from clinical outcomes, patients should be happy with other services you offer like short waiting time, support services, food and quality of communication. A simple way to know what patients want is by placing a suggestion box or through feedback surveys.

### Tracking patients with chronic conditions

Patients with conditions like glaucoma or diabetes need to be monitored regularly to preserve their vision. A patient register to track such patients and sending reminders via SMS or WhatsApp may be useful. You can track the effectiveness of such interventions through compliance to periodic follow-up.

### Benchmarking

Any evidence by itself in isolation does not give much insight. Looking at historical data or a comparison with other providers can give rich insight. For example, an indicator of quality of surgery is when a hospital reports that 80 per cent of cataract patients gained best corrected visual acuity (BCVA) 6/18 or better. When you compare this with WHO standards of 90 per cent you can find the gap and opportunities for improvement.

### Monitoring system

For excelling in operations, monitoring should be an integral part of a hospital teams work. Periodic systematic review of what went well or what didn't, can pave the way for continuous improvement of existing systems and processes.

## Conclusion

Managing demand has two facets and each has a different orientation. Looking at the current demand for services that is, those who come and have their need met by your services. All providers, regardless of whether you are programme manager or running a hospital, should also be concerned with those in the catchment area you are not reaching, the unmet need. Coverage is the percentage of those who have their need met out of all those who have a need. This needs to be based on good evidence generated through ongoing monitoring and population based studies.
